# Modified and Optimized Glass Electrode for pH Measurements in Hydrated Ethanol Fuel

**DOI:** 10.3390/molecules27228048

**Published:** 2022-11-19

**Authors:** Natalia Cambiaghi Atilio, Fernando Luis Fertonani, Elcio Cruz de Oliveira

**Affiliations:** 1 Postgraduate Programme in Metrology, Pontifical Catholic University of Rio de Janeiro, Rio de Janeiro 22451-900, Brazil; 2 Biosciences, Languages and Exact Sciences Institute (Ibilce), São Paulo State University (Unesp), Rua Cristovão Colombo, 2265, Jardim Nazareth, São José do Rio Preto 15054-000, Brazil; 3 Logistics, Operational Planning and Control, Measurement and Product Inventory Management, PETROBRAS S.A., Rio de Janeiro 20231-030, Brazil

**Keywords:** ethanol pH, hydrous ethanol fuel, pH indicators, chemometrics, modified and optimized glass electrode, metrology

## Abstract

One of the quality control parameters of ethanol fuel is pH, established by the Brazilian standard ABNT NBR 10891, whose scope is specific for hydrated ethanol fuel, and by the American standard ASTM D 6423, which focuses on anhydrous ethanol fuel. This study presented a modified and optimized structure using a single solvent, both for the glass electrode and the external reference electrode, to minimize the presence of the liquid junction potential for measuring the pH of hydrated ethanol fuel. The Box–Behnken design enabled us to determine the optimal condition expected for the new measurement system, which was compared with the systems proposed by the standard references and the turning range of acid–base indicators using parametric and nonparametric tests. The results revealed that the pH values obtained by the different systems are statistically different, and that only the values obtained by this proposal are suitable for the pH range found by the indicators. The optimized electrode presented an adequate response sensitivity to the Nernst equation, having an operational behavior adequate for the modified and optimized glass electrode for pH measurements in hydrated ethanol fuel.

## 1. Introduction

In 2019, renewable energy sources, which include bioenergy, wind, hydraulic, and solar energy, reached a 46.3% share in the Brazilian energy matrix, with 7.0% represented by ethanol fuel. However, with water scarcity in 2021, combined with the impact of the COVID-19 pandemic, the share of renewable energy in the energy matrix reduced to 44.7%. Consequently, the use of ethanol followed this trend, which is now 5.9% [1]. 

Brazil is a major producer of ethanol; evidence of this is that, in August 2020, the country exported 341.73 million liters, the largest export made in the last seven years [2]. For export to be possible, the product must be within the required quality control parameters. After an extensive literature search, a gap was found in the literature measuring the pH of ethanol, as the current concept applies only to aqueous solutions [3].

The potentiometric technique for pH determination is based on hydrogen ion activity, whose assumptions are normally applicable to aqueous solutions. To be considered an aqueous matrix, it must have at least 30% water in its composition. This concept does not apply to ethanol-in-water mixtures because hydrated ethanol fuel (HEF) has a maximum of 7.5% water (m/m). Thus, pH measurements that are carried out on samples of ethanol, hydrated ethanol fuel (HEF), can present several problems, such as (i) the instability of the readings; (ii) the need for long response times; (iii) results dependent on the type of electrode used. These problems are probably due to low solvent conductivity, dehydration of the glass electrode membrane, measurement time, and development and variation of the liquid junction potential (E_lj_) [4,5,6,7]. This liquid junction potential can develop at an interface, which occurs at its diaphragm in the case of the combined glass electrode. The E_lj_ developed in the diaphragm results from the balance of liquid systems (water/water; water/ethanol; ethanol/ethanol). This potential can occur when the ionic species present in the solutions have different mobility values, ionic acid, or, when dealing with the same electrolytes, but with different concentrations. This difference in mobility or ionic concentration promotes the separation of charges at the interface, thus generating the net junction potential (E_lj_). The development of this potential, which can assume a positive or negative value, is added to the global potential of the cell, compromising the measurement results.

The current structure in the construction of the electrode can lead to the development of the liquid junction potential in the diaphragm region, thus affecting the results of the calibration/adjustment of the measurement system, which compromises the measurement results. A reliable calibration procedure requires that the pH buffer solutions used for calibration/adjustment in ethanolic medium (HEF) be produced with the same solvent that makes up the sample [8,9,10].

In 2020, the Brazilian Energy Research Company (EPE) released data indicating a reduction in oil derivatives in the country’s final energy consumption, with a projected reduction of more than 2% by 2029. The justification for this forecast lies in the reduction of the growing supply of natural gas and biofuels [11].

At present, ethanol is one of the most used biofuels, and to guarantee its efficiency and its production, it must meet the quality requirements established for national trade and exports. Thus, this work focused on studying one of the quality requirements, the pH of hydrated ethanol fuel.

The quality of ethanol fuel is essential to guarantee the good performance of an engine. The presence of contaminants or irregular pH can cause the formation of deposits on metallic parts, clogging of filters, injectors, and fuel pumps, as well as the wear and corrosion of engine parts due to the formation of hydrolyzed species of iron ions (Fex(OH)y and polymerized ones) [12]. Another important aspect of the pH effect is the stress corrosion cracking enhanced by water and chemical species (chloride, oxygen, and acetic acid) at the steel–ethanol interface due to the synergistic action.

The pH of ethanol is one of the parameters used to evaluate the fuel quality, being established in Brazil by ABNT NBR 10-891:2018 and internationally by ASTM D 6423:2020. However, such documents have contradictory requirements, as they use different systems to perform the measurement. The main difference between these standards is in the electrode composition, corroborated by comparative studies that have shown that the values obtained are significantly different [8]. The ASTM D 6423:2020 standard is applied to anhydrous ethanol fuel, which uses a specific Orion electrode, Ross Sure-Flow, with an internal solution of potassium chloride in an aqueous medium [9]. The use of the ABNT NBR 10891 standard is for hydrated ethanol fuel. The pH of this compound is measured using a combined glass electrode with the external electrode filled with a lithium chloride solution in an ethanolic medium; however, the internal solution of the glass electrode is still aqueous [10]. This difference in the composition of the solutions can cause distortions in the pH measurement results. The difference in the composition of the solvents is also present in the calibration process of the measurement system used to perform the pH readings as the Certified Reference Material (CRM) used is prepared in an aqueous medium. For the calibration to be reliable, this study suggested that the pH calibration buffer solutions be produced with the same solvent as the sample [13].

The modification in the composition of electrodes aimed to improve the capacity and selectivity in detecting measurands of interest and is present in several areas and analytical applications. Several studies were found in the literature proposing different modification methods for this purpose, such as a modified glassy carbon electrode for the analysis of toxic agents present in pharmaceuticals and the food industry [14,15], modified biosensors for the detection of ethanol as an organic compound toxic and volatile in simulated body fluid [16], nitrogen-doped graphene electrode for the detection of an antimicrobial agent frequently used in personal care products [17], and a platinum electrode modified with Mn_3_O_4_ and chitosan for the simultaneous detection of selenium and nickel in an aqueous medium [18].

Therefore, this study aimed to optimize a modified glass electrode for measuring the pH of hydrated ethanol fuel that has hydrated ethanol as the only solvent, i.e., the same solvent in the internal (buffer of lithium acetate in acetic acid) electrode and external electrode (LiCl in ethanol) to obtain a new paradigm, where the glass electrode and external electrode have the same composition in terms of ethanol. The external electrode being composed of ethanol/LiCl allows to minimize the undesired effect of the liquid junction potential. Next, four different pH indicators using the turning range in an ethanolic medium were used as a reference system for the measurement of pH in ethanol. Finally, the response sensitivity of this new structure to the Nernst equation was evaluated.

## 2. Materials and Methods

**Figure 1:**
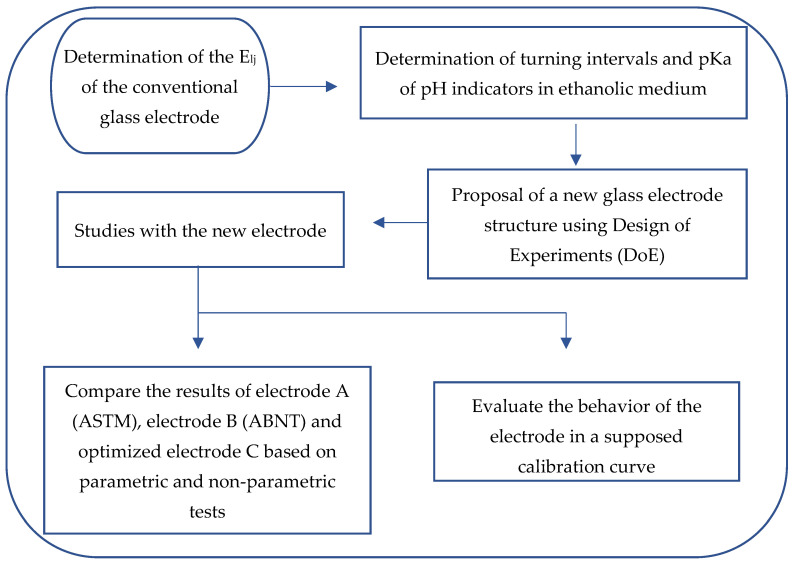
Experimental flowchart.

For a better understanding of this section, an experimental flowchart was described, Figure 1.

### 2.1. Determination of pKa in Ethanolic Medium

To assess whether the acid–base indicators have different behaviors with the variation of the aqueous or ethanolic medium, experiments were carried out to determine the pKa values for different indicators in an ethanolic medium.

An amount of 200 μL of the indicator was added to 100 mL of a solution of hydrated ethanol, 93.5% (m/m). Subsequently, increasing volumes (10 μL) of acid and/or base (HCl and/or NaOH 1.0 mol L^−1^) were added to the solution containing the indicator for the initial pH measurement and adjustment.

The pH of the solution was obtained using a conventional glass electrode and an external electrode filled with a saturated solution of KCl in water. For this electrode, the liquid junction potential was experimentally determined (E_lj_) and, later, corrected to obtain the pH value in ethanolic solution.

From the color variation of the indicator, with the addition of the acid or base solution, some aliquots were removed, and their absorption spectra were obtained in 350 nm ≤ λ ≤ 600 nm. The peak intensities shown by absorption wavelengths spectra were obtained for the acidic and basic forms of the dye. This experiment was applied to all indicators used: bromocresol green, methyl red, xylenol orange, and bromophenol blue. From the collected data were obtained the figures of pH (*Y*-axis) versus -log [HIn]/[Ind^−^] (*X*-axis) in accordance with Equation (3) (Henderson–Hasselbalch). This experimental procedure was applied to all indicators used: bromocresol green, methyl red, xylenol orange, and bromophenol blue.

The mean value of E_lj_ was carried out using a pair of silver chloride electrodes, previously tested for the potential difference (0.000 ± 0.002) mV, for T = 22 °C: (i) The external electrode to the combined glass electrode (composition: aqueous solution saturated with KCl); (ii) A reference electrode filled with 3.0 mol L^−1^ ethanolic lithium chloride (LiCl) solution, immersed in an HEF solution.

A digital multimeter with five-and-a-half digits was used for E_lj_ measurements. The measurement was performed over five days, and the mean value obtained was E_lj_ = 24.58 mV. To convert this E_lj_ value into pH, Equation (1) [3] was applied:

(1) $$\alpha \;=\frac{\Delta U}{\Delta \mathrm{pH}}$$

**Figure 2:**
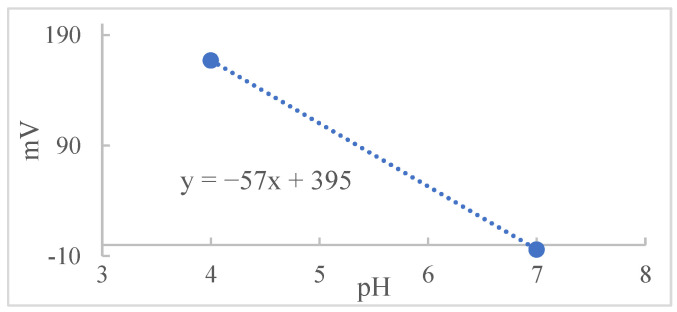
The pH value versus mV obtained by the pH meter calibration system.

From the Nernst equation, α is the slope, ΔU is the value of the measured potential change, i.e., E_lj_, and ΔpH is the pH range corresponding to ΔU. Dividing the potential value by the angular coefficient obtained from the calibration of the pH meter, Figure 2, the result of the quotient found is the value of ΔpH. This value should be discounted from the pH value obtained during the experiments [5,19,20].

### 2.2. Construction of the New Structure for the Glass Electrode

The new structure for the electrode was based on the use of a single solvent, hydrated ethanol fuel, both for the glass electrode and the external reference electrode.

Based on the great experience of authors in the field of electrochemistry and from the variables (factors) and their respective levels, the Box–Behnken design (BBD) was chosen to study the contribution of ionic strength, buffer solution concentration, and temperature to constructing a new glass electrode for pH measurement in HEF, Table 1.

**Figure 3:**
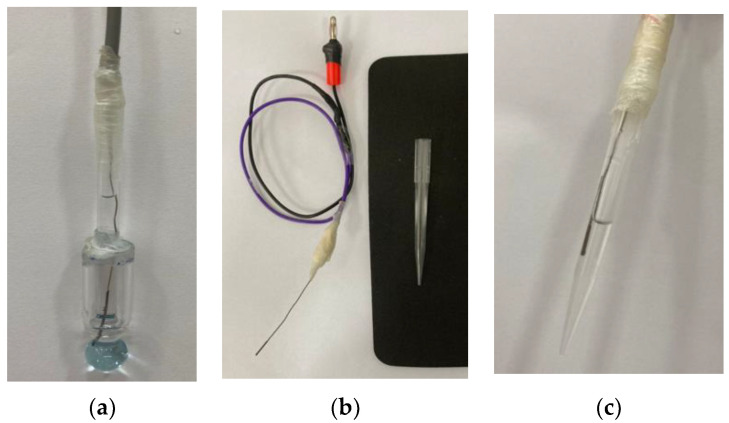
Modification of the electrodes: (**a**) Internal electrode completed; (**b**) Mounted electrode showing the weld region and micropipette tip used as the body for the external reference electrode; (**c**) Finished external electrode and filled with a solution of an adequate concentration of LiCl.

The new structure was prepared from a universal combined glass electrode of the brand Sensoglass, model SC09. With the aid of a glass cutter (diamond), the external body of the combined glass electrode was removed. The internal solution was removed and replaced with pure water. The internal electrode, after changing the solution, was used to maintain the original configuration of the Ag/AgCl electrode system, Figure 3a. The construction of the reference electrode was performed using the Ag/AgCl electrode previously removed from the glass electrode (external electrode). The same Ag/AgCl electrode was used to maintain the original pair of electrodes of the combined glass electrode. Figure 3b,c show the preparation steps and the finished external Ag/AgCl and LiCl in the HEF electrode, respectively. This electrode does not have a diaphragm to minimize the presence of the liquid junction potential and the tip was filled with a solution of lithium chloride in ethanol (HEF). This versatility presented by the tip electrode facilitated the exchange of the internal solution for others with different values of ionic strength.

Finally, the internally modified glass electrode and the reference electrode were connected to the pH meter and the thermometer to control the temperature.

### 2.3. Design of Experiments (DoE)

The experiments with the modified electrode were conducted randomly, so all possible combinations from the different levels of variables were considered.

To compare the performance of the optimized electrode, a 0.050 molal buffer solution was used. The pH and potential in mV of the buffer solution were measured using the conventional electrode filled with KCl saturated in water (electrode A), the electrode suggested by ABNT (electrode B), and the electrode optimized by BBD (electrode C) for this purpose.

Another comparison was with the indicators, considered as a reference from their turning ranges previously obtained in HEF. The turning ranges of the indicators were obtained by graphical extrapolation after experimental determination of pKa values in HEF.

The first part introduces the experimental design, and the Box–Behnken design (BBD) was used, considering one replicate for each factorial level and three replicates at the center point. This type of design has great potential for use in several areas, especially when there are three variables with a quadratic fit, such as micellar liquid chromatography in food samples [21]; in bean seed samples using ICP OES [22]; in the textile industry [23]; in the pharmaceutical research [24]; in the bioethanol production [25,26]; in routine analysis in seafood [27] and edible natural pigments [28].

The second part was a review and description of the parametric and nonparametric statistical tests used in this study, covering the Shapiro–Wilk normality test, the tests for detecting outliers, and finally, the tests for comparing samples [29].

### 2.4. Measurements Using the Three Electrodes

Independent samples of the buffer solution in an ethanolic medium (0.050 molal) were subjected to pH measurement over ten weeks, a period when all the experimental parts were carried out; therefore, the stability of the solutions was guaranteed. The pH readings were obtained every Tuesday, at the same time interval, in the morning, with three repetitions (n = 3, independent samples). The measurement values obtained for the three different electrodes are shown in Table 2, together with the respective potential values in mV provided, in parentheses, simultaneously with the pH values by the pH meter.

To evaluate the behavior of the indicators in an ethanolic medium, HEF solutions were used and 200 µL of the methyl red indicator was added to each solution. After adding the indicator, microvolumes of solutions of known concentrations of acid and base were added. Additions were carried out in two independent experiments: (i) 0.1 mol L^−1^. hydrochloric acid (HCl) solution, to evaluate the spectrophotometric behavior of the indicator when the acid species is present; (ii) the same procedure was applied to evaluate the basic species of the indicator, using a solution of sodium hydroxide (NaOH) 0.1 mol L^−1^. The evaluation of the spectrophotometric behavior of each species, acidic or basic, was performed by observing the maximum absorbance peak points (λ_max_) for each of the solutions. Finally, two aliquots of the buffer solution (BS) were taken, where one aliquot was acidified with the addition of 1.0 mol L^−1^ HCl, H^+^ and the other aliquot was basified with the addition of 1 mol L^−1^ NaOH, OH^−^, causing the BS purposefully to lose its buffer capacity so that it was possible to perform the reading using the electrode C at different pHs and evaluate its behavior at 25 °C (Table 3). This study was carried out to evaluate the behavior of the electrode in a supposed calibration curve.

## 3. Results and Discussion

### 3.1. Determination of pKa in Ethanolic Medium

After experimenting with the methyl red indicator, the pH values of each withdrawn aliquot were corrected by applying Equation (1) (see Section 2.1); $-57.0=\frac{24.58}{\Delta \mathrm{pH}}$.

We found a value of ΔpH equal to −0.43, close to the one presented by Battes for the mixture of ethanol and 90% water, −0.40. For each pH value measured by the conventional glass electrode, 0.43 must be discounted to obtain the pH value corrected for the effect of the liquid junction potential, as shown in Table 4 [19].

**Figure 4:**
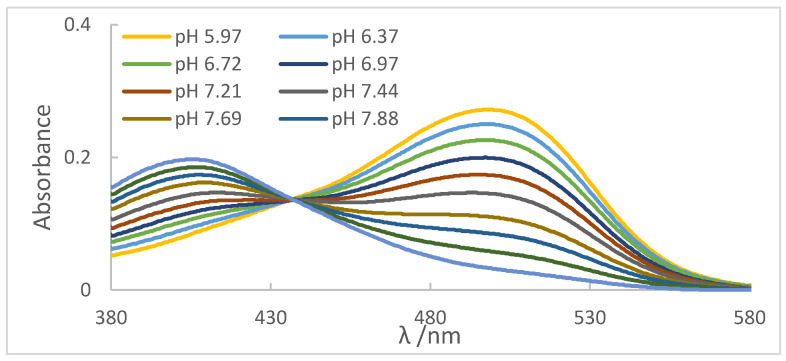
Spectra obtained for each of the aliquots using the methyl red indicator.

For each aliquot of the ethanolic solution containing the indicator, the reading was performed on the spectrophotometer, as shown in Figure 4. It can be observed the absorbance peaks (A_maximum_) present at λ = 413 nm, referring to the basic species of the indicator, which presented a yellow color, and the peaks presented at λ = 510 nm, referring to the acidic species of the indicator, whose color was red. Another important aspect was the presence of the isoabsorbed point (isobestic); that is, the wavelength at which all absorbing species had the same absorptivity, so Beer’s law was not affected by equilibrium at λ = 438 nm, evidencing the presence of two species in equilibrium.

**Figure 5:**
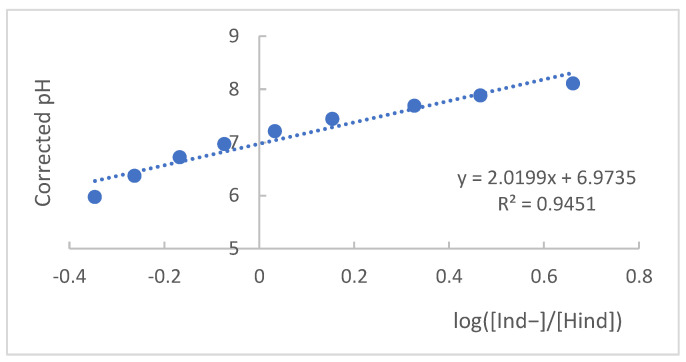
Graphical representation of pH values as a function of log value ([Ind−]/[Hind]).

To find the *pKa* value, the spectrophotometric principles presented above were applied. From the collected absorbance values and the maximum absorption wavelengths, for the acidic and basic forms of the indicator, it was possible to apply the mathematical expression presented in Equation (2) (Henderson–Hasselbalch equation) [30] and its graphical representation of the log ratio [A_Ind−_/A_Hind_] as a function of corrected pH, as shown in Figure 5 [19,31].

(2) $$\mathrm{pH}=\mathrm{pKa}+\log\frac{\left[\mathrm{Ind}-\right]}{\left[\mathrm{Hind}\right]}$$

Starting from Equation (2) and graphically representing the corrected pH values as a function of the values of $\log\frac{\left[\mathrm{Ind}-\right]}{\left[\mathrm{Hind}\right]}$, as shown in Figure 5, it was possible to find the value of pKa from the intercept of the curve with the Y-axis, a situation in which pH = pKa, which, for the methyl red indicator, is equal to 6.98.

**Figure 6:**
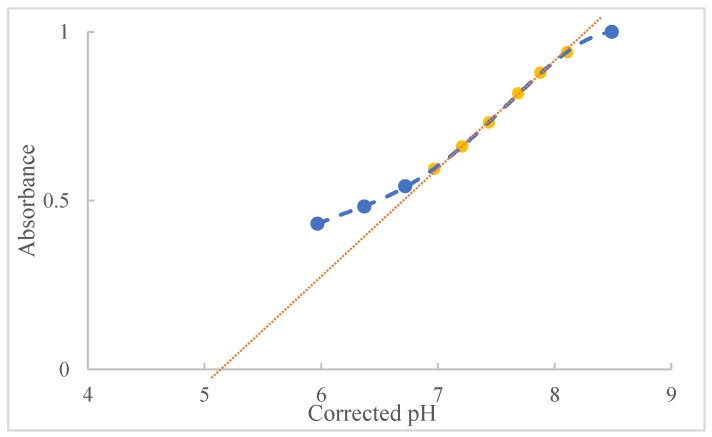
Graphical representation of the values of absorbance versus pH for the determination of the values of the turning range of the methyl red. In yellow are the points that present the best correlation and were used to perform the extrapolation of the curve.

To determine the turning interval, in addition to the careful observation of the operator to reveal the interval, it was also accompanied by graphically obtaining the beginning and end values of the indicator turning process. In Figure 6, the normalized absorbance values for the indicator as a function of the corrected pH are shown. The extrapolation obtained from the dotted curve provides the lower and upper pH limits for the turning of the indicator, where the points in yellow were the ones that obtained the best correlation, 99.93%. Consequently, these will be the points used to perform the curve extrapolation [19].

Using the Excel statistical software, based on analysis of variance, both linear regression and the intercept of the calibration plot were significant (*p* < 0.05). Therefore, the value of the intercept (linear coefficient), −1.5701, and the slope of the line, 0.3099, were found using an unweighted linear fit, Equation (3), where *y* represents absorbance and *x* is pH.

(3) y=βx+α

where *α* represents the linear coefficient and *β* the slope, we obtain the following Equation (4):

(4) A=0.3099 pH−1.5701

To determine the values of the extrapolation of the line, that is, where it crosses the *y -axis* at 0 and 1, as they are normalized values, using Equation (4), and solving for pH, Equations (5) and (6):

For *A* = 0:

(5) 0=0.3099 pH−1.5701

pH=5.06

For *A* = 1:

(6) 1=0.3099 pH−1.5701

pH=8.29

Thus, it was possible to determine the values of the indicator turning interval for the ethanolic medium, which in the case of methyl red starts at 5.06 and ends at 8.29, with a pKa of 6.98. This procedure was carried out for the other indicators used, obtaining new pKa and pH values from their respective turning intervals. The equation obtained for each of the indicators and the new range values are described in Table 5.

### 3.2. Construction of the New Glass Electrode

Based on the results of the experiments in Table 6, it was noticeable that two experiments (9 and 10) reached values very close to each other as well as to the expected value. Analyzing these two experiments, it was observed that only the level of variable 3, corresponding to temperature, was changed. The same occurred when we compared experiments 7 and 8, and 11 and 12, showing that when the value for variables 1 and 2 was maintained and the value of variable 3 was changed, the results were very similar. This behavior demonstrated that variable 3 (temperature) was not statistically significant for the experiment, besides the *p*-value being higher than 0.05. Otherwise, the results of the cited experiments would present a more significant variation between them.

When comparing experiments where the levels of variables 1 and 3 were maintained, the value of variable 2 (ionic strength) is altered, evidencing that variable 2 had great relevance for the experiment. The variable 1 (buffer solution composition) had great relevance for the experiment due to the great difference in the results due to its level changing.

After evaluating the results, the optimized condition was used in experiment 9, where a buffer solution with a concentration of 0.050 molal, ionic strength of 0.1 molal, and temperature of 20 °C was used. Although the temperature was not relevant to the experiment, we chose to keep the temperature at 20 °C for the experiments in the next section.

### 3.3. Studies Carried out with the Optimized Electrode

As mentioned before, to correct the net junction potential, it is necessary to deduct 0.43 from each pH value measured by electrode A. This value was experimentally determined by measuring the value of the net junction potential (E_lj_) established between the internal and external region of the reference electrode and later converted to the respective value expressed in pH, as discussed above. The corrected values for electrode A are shown in Table 7 and are used from now on for electrode A.

For a significant level of 0.05, and based on parametric and nonparametric tests, it was possible to evaluate and compare the results from electrode A (ASTM), electrode B (ABNT), and the optimized electrode C, Table 8.

**Figure 7:**
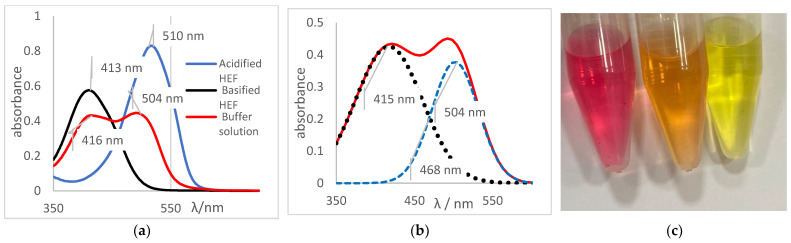
(**a**) Spectrum of the methyl red indicator. Blue: acidified HEF solution. Black: basified HEF solution. Red: buffer solution; (**b**) Deconvolution of the buffer solution curve; (**c**) Visual perception.

The determination of the pH values of different aqueous solutions using acid and basic indicators was the first method used to determine this important parameter, before the development of potentiometers and glasses electrodes. Thus, for this reason the method was adapted and applied for the determination of the pH and pKa of the indicators prepared in an ethanolic solution of HFE. The concept of Henderson–Hasselbalch was applied, considering that indicators as defined as weak organic bases or acids and, for the most part, are soluble in ethanol. Thus, for this research, a reference system was prepared using a set of ethanol-soluble indicators. This set was applied to generate a reference system for pH measurement in ethanol independent of any electronic system composed of a pH meter and glass electrode. The pH values of the ethanolic solutions were determined by applying the turning ranges of each indicator to different aliquots of the ethanolic solution (HEF) as shown in Table 5. Methyl red indicator was used as an example for others due to its impeccable physicochemical behavior. The spectrum shown in Figure 7a was obtained by the methyl red indicator for an acidified HEF solution, a blue curve; a basified HEF solution, a black curve; and for the ethanolic buffer solution, a red curve, which was intended to determine its pH value.

The spectral behavior analysis was based on observing the values of maximum λ of absorbance in the bands of each solution, as shown in Figure 7a. The acidified and basified solutions had maximum absorbance peaks at 510 nm and 413 nm, respectively. The presence of two bands, simultaneously, was noticeable, showing that the pH of the solution is between its turning range, which in this case was from 5.06 to 8.29 (experimental data available on Table 5), with an average pH value close to 6.67. It should be noted that the spectral behavior of the buffer solution differed from the other indicators (acid or basic) concerning the isoabsorptive point [32,33]. This statement was ratified through the analysis of the curve deconvolution, using Software Origin 7.0, in which the presence of the basic (415 nm) and acidic (504 nm) species was clearly demonstrated, as shown in Figure 7b. It was noticeable that the color of the tube that contained the sample of buffer solution presents an orange color, as shown in Figure 7c., the result of the mixture of the yellow color, the basic species, with red, the acidic species, thus showing the presence of the two species of the indicator; that is, the sample pH was close to the average value of the used indicator range, which is 6.67, which corroborated the spectral behavior discussed earlier.

Table 9 presented the spectra obtained, and the visual results of the experiments carried out simultaneously. The colors developed by the dyes (pH indicators) were observed for the same experimental conditions in test tubes for visual comparison. Falcon tubes can be seen from left to right, respectively: (i) the acidified HEF solution; (ii) the buffer solution to be determined for pH, and (iii) the basified HEF solution.

For the xylenol orange, the presence of the acid species, with a maximum at λ = 433 nm, and of the basic species, with a maximum at 588 nm, was perceptible. When the behavior of the buffer solution was analyzed, its similarity in behavior with the acidic species was evident due to the partial overlap, which is what indicated that the pH of the buffer solution was below the acid turning range of this indicator, as shown in Table 5; that is, the pH of the solution is less than 6.96. Furthermore, the visual perception showed that the buffer solution was very similar to the acidified solution, with a yellow color, thus assuming it agreed with the spectral result, a pH ≤ 6.96.

Next, for the bromophenol blue, the presence of two bands very close to the peaks of the acidic and basic species was visualized. However, the peak at 602 nm was noticeably higher; that is, it had a higher absorbance. The visual inspection for the buffer solution was bluish green, showing a greater contribution of the basic species (a blue tone) than the acid species, which had a yellow color. From these results, it was possible to infer that the pH of the solution must be above the middle of the range, 6.18, and below the basic range, 7.22, as shown in Table 5.

Finally, for the bromocresol green, the presence of two bands was visible, indicating the presence of acidic and basic species. Thus, considering the equivalence of the absorbance of the acid and basic species, the pH of the solution must be close to the middle of the range, as shown in Table 5, with a pH = 6.72, a value very close to that found by the methyl red (6.67). The same result was obtained in the visual perception, where the coloring of the buffer solution presented a shade of green (tube in the middle), evidencing the mixture of a blue and yellow color.

The pH results obtained by the indicators, investigated into the ethanolic buffer solution, considering the spectra and visual inspection, were grouped, as shown in Table 10. The most relevant results were obtained by the methyl red and bromocresol green indicators, as both reveal that the pH in the middle of their ranges and values are very similar. From these results, it was possible to narrow the pH range of the buffer solution, indicating that the pH of the solution is very close to the range from 6.67 to 6.72.

Using the pH range determined using the pH indicators, it was possible to compare the results obtained by the three different electrodes, A, B, and C. As mentioned before, the distributions of electrodes A and B were Gaussian; therefore, the mean value was used. On the other hand, for the electrode C, the median was used, as shown in Table 11.

Comparing the mean and median values, as shown in Table 11, with the values of the pH intervals found by the indicators, it was noticeable that the closest was the value obtained by the median of the distribution of electrode C. It was even very close to the values found by methyl red and bromocresol green, thus demonstrating efficiency in the measurement using the optimized electrode.

This study using the pH indicators found a pH for the buffer solution close to the range from 6.67 to 6.72. When comparing the mean values obtained for electrodes A and B, 6.48 and 6.59, respectively, and the median value for electrode C, 6.71, it was evident that the result that best fitted the indicators was the result obtained by electrode C.

**Figure 8:**
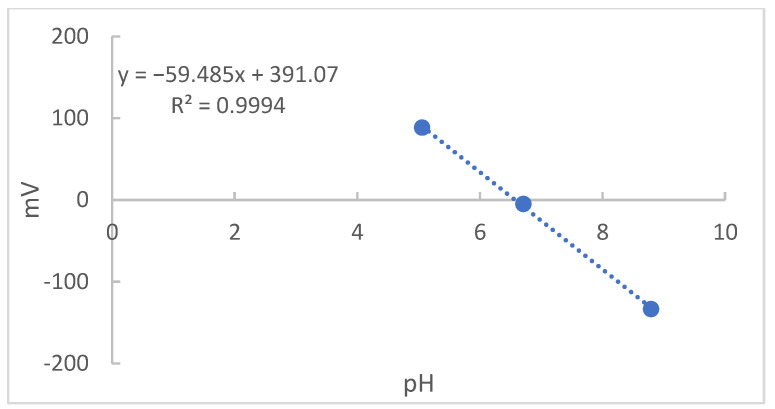
The pH values versus mV obtained for the different solutions from electrode C.

Finally, the values found in Table 3 were graphically recorded in Figure 8. Since the angular coefficient of the regression was 59.48, a value referring to 100.5% of Nernst’s ideal response, 59.16 to 25 °C, it was an indication of an adequate sensitivity of the optimized electrode [19].

## 4. Conclusions

A modification of a commercial glass electrode was carried out using hydrated ethanol fuel, changing the paradigm of measurements performed in a mixed aqueous system (glass electrode filled with water solutions and external as KCl/water or LiCl/water) to a fully ethanolic system. From the BBD matrix investigation considering the three variables studied, each one at three different levels, it was possible to determine the optimized working condition: the concentration of the buffer solution (0.050 molal), ionic strength (0.10 molal), and the temperature was not statistically significant.

After a preliminary evaluation for this new structure for the glass electrode applying colorimetric indicators as the reference system to the pH measurements in ethanol solutions, the results showed that the optimized electrode presented an adequate response and sensitivity to the Nernst equation (59.48 mV per decade), having an operational behavior adequate to the new measurement system proposed by the present study.

For future research, it is recommended: (i) Continuing the study of the optimized electrode to evaluate its operation over time of use, stability, sensitivity, and robustness, together with possible ways of storage, to prolong its useful life and guarantee the quality of measurements; (ii) Using different types of ethanol for the comparative analysis between the pH measurements; (iii) Elaborating and deepening the study on a possible candidate for pH certified reference material in an ethanolic medium for application in the calibration of the measurement system using the optimized electrode for the pH measurement of ethanol fuel.

It is worth noting that the improvement of the optimized electrode and the development of a candidate for Certified Reference Material constitute a proposal for a PIPE-FAPESP project (Innovative Research in Small Businesses) in partnership with Empresa SPR Soluções Metrológicas LTDA, with the feasibility of creating a possible spin-off for this specific product line.

## Figures and Tables

**Table 1:** Levels of variables chosen for BBD.

Variables	Symbol	Coded Variable Level		
		Low	Central	High
		−1	0	+1
* Buffer solution (molal)	X_1_	0.001	0.050	0.100
** Ionic strength (molal)	X_2_	0.1	1.6	3.1
Temperature (°C)	X_3_	20	25	30

* Lithium acetate in acetic acid (composition of 1:1). ** Solution of lithium chloride in ethanol.

**Table 2:** Results obtained by electrodes A, B and C, n = 3.

Week	pH								
	Electrode A			Electrode B			Electrode C		
1	6.82 (9)	6.93 (3)	6.96 (1)	6.49 (40)	6.42 (43)	6.40 (44)	6.67 (−2)	6.69 (−4)	6.71 (−5)
2	6.89 (5)	6.91 (4)	6.87 (5)	6.63 (31)	6.63 (31)	6.63 (31)	6.71(−5)	6.72 (−6)	6.71 (−5)
3	6.80 (9)	6.86 (6)	6.84 (7)	6.41 (43)	6.47 (40)	6.50 (39)	6.74 (−7)	6.72 (−6)	6.72 (−6)
4	6.89 (4)	6.91 (3)	6.93 (2)	6.68 (28)	6.67 (29)	6.71 (27)	6.63 (0)	6.63 (0)	6.64 (−1)
5	6.94 (1)	6.96 (0)	6.94 (1)	6.40 (45)	6.35 (47)	6.35 (47)	6.67 (−2)	6.65 (−1)	6.67 (−2)
6	6.84 (7)	6.93 (2)	6.89 (4)	6.57 (34)	6.61 (32)	6.60 (33)	6.66 (−2)	6.70 (−4)	6.67 (−3)
7	6.92 (2)	6.86 (6)	6.81 (9)	6.54 (36)	6.56 (35)	6.57 (34)	6.72 (−5)	6.71 (−4)	6.72 (−5)
8	6.84 (7)	6.87 (5)	6.96 (0)	6.88 (17)	6.87 (18)	6.88 (17)	6.73 (−7)	6.75 (−8)	6.72 (−5)
9	6.94 (1)	6.96 (0)	6.94 (1)	6.76 (24)	6.78 (23)	6.78 (23)	6.72 (−5)	6.70 (−4)	6.70 (−4)
10	7.03 (−4)	7.03 (−4)	6.98 (−1)	6.56 (36)	6.57 (34)	6.56 (35)	6.73 (−6)	6.72 (−6)	6.72 (−5)

Values inside the ( ) are in mV.

**Table 3:** Values obtained by reading the solution and acidified and basified solutions using electrode C.

	Solutions		
	H^+^	BS	OH^−^
Potential	88.43 mV	−5 mV	−133 mV
pH	5.05	6.71	8.79

**Table 4:** The pH values obtained for each of the aliquots, using the methyl red, and their corrected values.

Aliquot	Read pH	Corrected pH	Aliquot	Read pH	Corrected pH
1	6.40	5.97	6	7.87	7.44
2	6.80	6.37	7	8.12	7.69
3	7.15	6.72	8	8.31	7.88
4	7.40	6.97	9	8.54	8.11
5	7.64	7.21	10	8.92	8.49

**Table 5:** Values of the turning range and pKa found for the ethanolic medium 93% (m/m).

Indicator	Acid Range	pKa	Middle of the Range	Basic Range	Equation
Methyl red	5.06	6.98	6.67	8.29	A=0.3099 pH−1.5701
Bromocresol green	6.00	6.92	6.72	7.45	A=0.543 pH−3.1618
Xylenol orange	6.96	8.60	8.14	9.31	A=0.4262 pH−2.9701
Bromophenol blue	5.14	5.81	6.18	7.22	A=0.4810 pH−2.4746

**Table 6:** BBD matrix.

Exp	BS (Molal)	I (Molal)	T (°C)	E (mV)	Exp	BS (Molal)	I (Molal)	T (°C)	E (mV)
1	0.001 (−1)	0.1 (−1)	25 (0)	−166	***9***	***0.050 (0)***	***0.1 (−1)***	***20 (−1)***	***0***
2	0.001 (−1)	3.1 (1)	25 (0)	−59	***10***	***0.050 (0)***	***0.1 (−1)***	***30 (1)***	***−3***
3	0.100 (1)	0.1 (−1)	25 (0)	123	**11**	**0.050 (0)**	**3.1 (1)**	**20 (−1)**	**321**
4	0.100 (1)	3.1 (1)	25 (0)	303	**12**	**0.050 (0)**	**3.1 (1)**	**30 (1)**	**330**
5	0.001 (−1)	1.6 (0)	20 (−1)	−155	13	0.050 (0)	1.6 (0)	25 (0)	−92
6	0.001 (−1)	1.6 (0)	30 (1)	−222	14	0.050 (0)	1.6 (0)	25 (0)	−74
**7**	**0.100 (1)**	**1.6 (0)**	**20 (−1)**	**202**	15	0.050 (0)	1.6 (0)	25 (0)	−88
**8**	**0.100 (1)**	**1.6 (0)**	**30 (1)**	**201**					

The values in parentheses are the coded variables. BS is buffer solution, I is the ionic strength, T is the temperature, E is the potential.

**Table 7:** Corrected pH values obtained for electrode A.

Week	pH			Week	pH		
1	6.39	6.50	6.53	6	6.41	6.50	6.46
2	6.46	6.48	6.44	7	6.49	6.43	6.38
3	6.37	6.43	6.41	8	6.41	6.44	6.53
4	6.46	6.48	6.50	9	6.51	6.53	6.51
5	6.51	6.53	6.51	10	6.60	6.60	6.55

**Table 8:** Comparing results from electrodes A, B and C.

Statistical Test		Electrode A	Electrode B	Electrode C	Evaluation
Shapiro–Wilk	Calculated value	0.9660	0.9531	0.8942	Electrode A and B have Gaussian behavior, but electrode C does not
	Critical value	0.9270			
Grubbs	Calculated value	2.50	2.39	-	No outliers
	Critical value	2.75		-	
*t*-test: different variances *	Calculated value	3.89		-	Results obtained by electrodes A and B are statistically different ^‡^
	Critical value	2.02		-	
Interquartile range (IQR)	Calculated value	-	-	6.67/6.72	No outliers
	Critical value	-	-	6.59/6.80	
Friedman	Calculated value	*p* < 0.0001			Results obtained by electrodes A, B, and C are statistically different
	Critical value	*p* = 0.05			

* H_0_ must be rejected, so there is no equality between the variances. ^‡^ Based on Wilcoxon signed rank test, the null hypothesis H_0_ is rejected when comparing the distributions of electrodes, A and C, and B and C.

**Table 9:** Spectra and visual perception.

Indicator	Spectrum	Visual perception
Xylenol orange	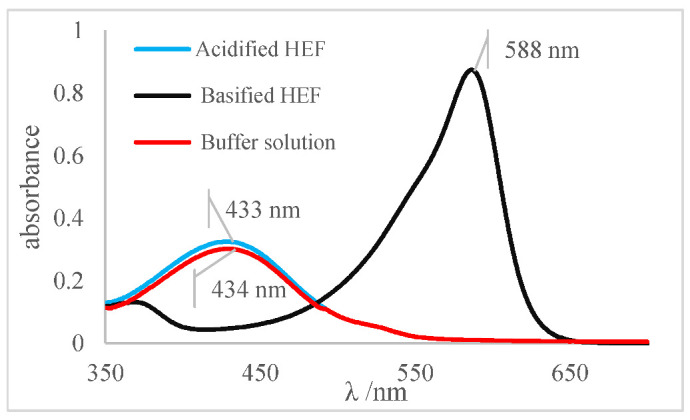	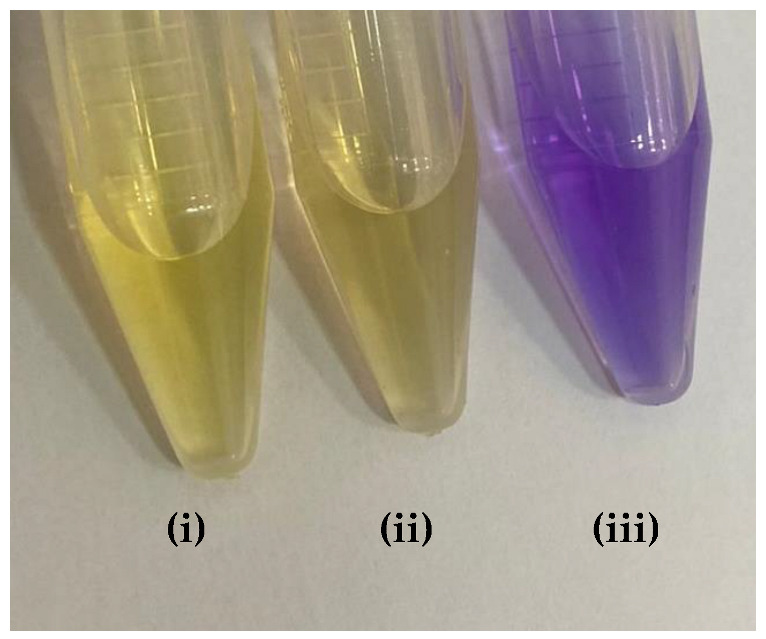
Bromophenol blue	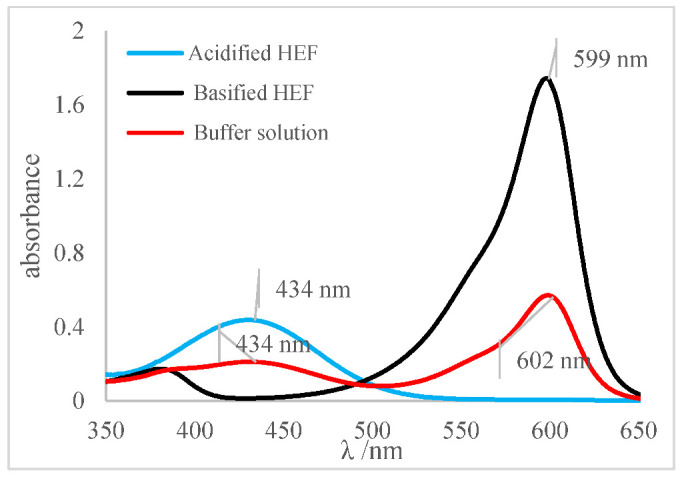	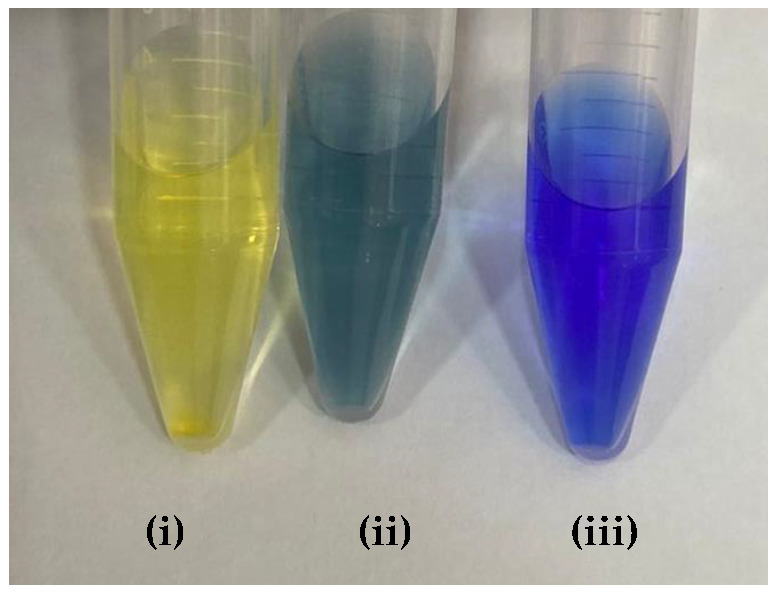
Bromocresol green	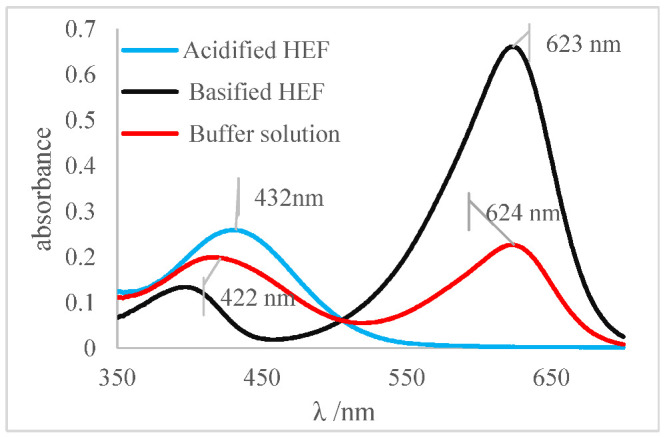	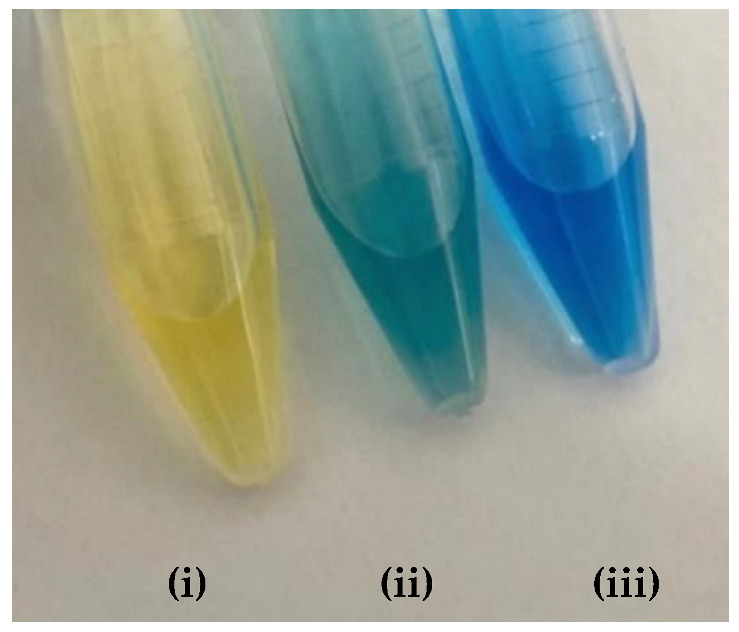

**Table 10:** Results of the pH values of the buffer solution using the indicators.

Indicator	pH
Methyl red	≈6.67
Xylenol orange	<6.96
Bromophenol blue	6.18 < pH < 7.22
Bromocresol green	≈6.72

**Table 11:** Values of the mean and median of the distributions obtained by electrodes A, B, and C.

	Electrode		
	A	B	C
Mean	6.48	6.59	-
Median	-	-	6.71

## Data Availability

Available data are presented in the manuscript.

## Sample Availability

Samples of the compounds are available from the authors.
